# Mesenchymal stromal cells promote liver regeneration through regulation of immune cells

**DOI:** 10.7150/ijbs.39725

**Published:** 2020-01-22

**Authors:** Chenxia Hu, Zhongwen Wu, Lanjuan Li

**Affiliations:** Collaborative Innovation Center for Diagnosis and Treatment of Infectious Diseases, State Key Laboratory for Diagnosis and Treatment of Infectious Diseases, First Affiliated Hospital, School of Medicine, Zhejiang University, Hangzhou, Zhejiang, PR China

**Keywords:** liver transplantation, mesenchymal stromal cell, liver regeneration, immunoregulation, anti-inflammation

## Abstract

The liver is sensitive to pathogen-induced acute or chronic liver injury, and liver transplantation (LT) is the only effective strategy for end-stage liver diseases. However, the clinical application is limited by a shortage of liver organs, immunological rejection and high cost. Mesenchymal stromal cell (MSC)-based therapy has gradually become a hot topic for promoting liver regeneration and repairing liver injury in various liver diseases, since MSCs are reported to migrate toward injured tissues, undergo hepatogenic differentiation, inhibit inflammatory factor release and enhance the proliferation of liver cells *in vivo*. MSCs exert immunoregulatory effects through cell-cell contact and the secretion of anti-inflammatory factors to inhibit liver inflammation and promote liver regeneration. In addition, MSCs are reported to effectively inhibit the activation of cells of the innate immune system, including macrophages, natural killer (NK) cells, dendritic cells (DCs), monocytes and other immune cells, and inhibit the activation of cells of the adaptive immune system, including T lymphocytes, B lymphocytes and subsets of T cells or B cells. In the current review, we mainly focus on the potential effects and mechanisms of MSCs in inhibiting the activation of immune cells to attenuate liver injury in models or patients with acute liver failure (ALF), nonalcoholic fatty liver disease (NAFLD), and liver fibrosis and in patients or models after LT. We highlight that MSC transplantation may replace general therapies for eliminating acute or chronic liver injury in the near future.

## Introduction

Early in 1931, it was demonstrated that the liver is a unique organ able to regenerate completely after removal of two-thirds of the liver mass in rats 1. The liver is composed of primary hepatocytes, cholangiocytes, Kupffer cells (KCs), liver sinusoidal endothelial cells, hepatic stellate cells (HSCs), fibroblasts, lymphatic vessel cells, oval cells, lymphocytes and other immune cells 2, 3. The cell death of hepatocytes and/or cholangiocytes subsequently triggers the activation of KCs, liver sinusoidal endothelial cells and HSCs 4, 5. Furthermore, other liver-specific immune cells, such as dendritic cells (DCs), natural killer (NK) cells, natural killer T cells (NKT) cells, and neutrophils, also respond to liver injury and subsequently produce cytokines and initiate anti-inflammatory responses 6. The blood supply of the liver moves through the hepatic artery and portal vein, which screen for systemic and gut-derived pathogens or toxins; thus, the liver is sensitive to pathogens that induce acute or chronic liver injury 7. At present, liver transplantation (LT) is still the only curative treatment for end-stage liver diseases 8, while the clinical application is limited by a shortage of liver organs, immunological rejection and high costs.

Mesenchymal stromal cell (MSC)-based therapy has gradually become a hot topic since MSCs are able to promote liver regeneration and attenuate liver injury after administration *in vivo*. MSCs can be isolated from multiple organs and tissues, including bone marrow, adipose tissue, peripheral blood, synovial membrane tissue, and cartilage 9. They are able to give rise to mesodermal lineages including adipocytes, osteocytes and chondrocytes 10. Moreover, transplanted MSCs take part in liver regeneration by migrating toward injured tissues, participating in hepatocyte differentiation and paracrine mechanisms and having immunomodulatory properties 11-13. In vitro culture with specific growth factors promoted the hepatogenic differentiation of MSCs into hepatocyte-like cells (HLCs) with liver-specific morphology and liver functions, including abilities to uptake low-density lipoprotein and indocyanine green, secrete albumin and urea, and store glycogen and the cells also had cytochrome P450 activity 14. *In vivo*, intrasplenic transplanted MSCs engrafted into carbon tetrachloride (CCl_4_)-treated liver tissues underwent hepatogenic differentiation into HLCs with typical hepatocyte morphology and formed a three-dimensional architecture 15. Furthermore, engrafted MSCs that had underwent hepatogenic differentiation further attenuated hepatocyte necrosis and promoted liver regeneration, subsequently improving the survival rate of ALF models after transplant into injured liver tissue 16. Administration of HLCs before partial hepatectomy significantly downregulated lipid accumulation and hepatocyte apoptosis and improved the survival rate of ALF animal models 17. However, Di Bonzo et al. showed that engrafted MSCs rarely undergo hepatogenic differentiation and disappear from the liver after injection within one month 18. MSCs are able to synthesize various growth factors and cytokines to exert paracrine effects in liver tissues to promote liver regeneration. MSCs secrete a large number of antiapoptotic growth factors, such as stromal cell-derived factor-1, vascular endothelial growth factor, hepatocyte growth factor and insulin-like growth factor-I, to prevent HSC activation and subsequent liver fibrosis 19. Moreover, MSC-derived growth factors such as hepatocyte growth factor, fibroblast growth factor, interleukin (IL)-6, fibrinogen and transforming growth factor (TGF)-α also participate in liver regeneration by promoting hepatocyte proliferation 20. They also decrease the expression of pro-inflammatory factors, including tumor necrosis factor-α (TNF)-α, interferon (IFN)-γ and IL-1β, and the expression of chemokines, including CXCL1 and CXCL2, *in vivo* to attenuate liver inflammation 21, 22. The paracrine mechanisms of MSCs in liver diseases are reviewed elsewhere 23. Interestingly, the immunoregulation of MSCs has gradually drawn attention since they effectively inhibit the activation of innate immune cells and activate cells of the adaptive immune system, including T lymphocytes, regulatory T cells (Tregs), T helper cells, B lymphocytes and regulatory B cells (Bregs). Consequently, MSCs generate a tolerogenic environment for maintaining immune homeostasis *in vivo*. In the current review, we mainly focus on the potential effects and mechanisms of MSCs in inhibiting the activation of immune cells to attenuate liver injury in models or patients with acute liver failure (ALF), nonalcoholic fatty liver disease (NAFLD), and liver fibrosis and in patients or models after LT.

## Liver regeneration and immune cells

**Figure 1:**
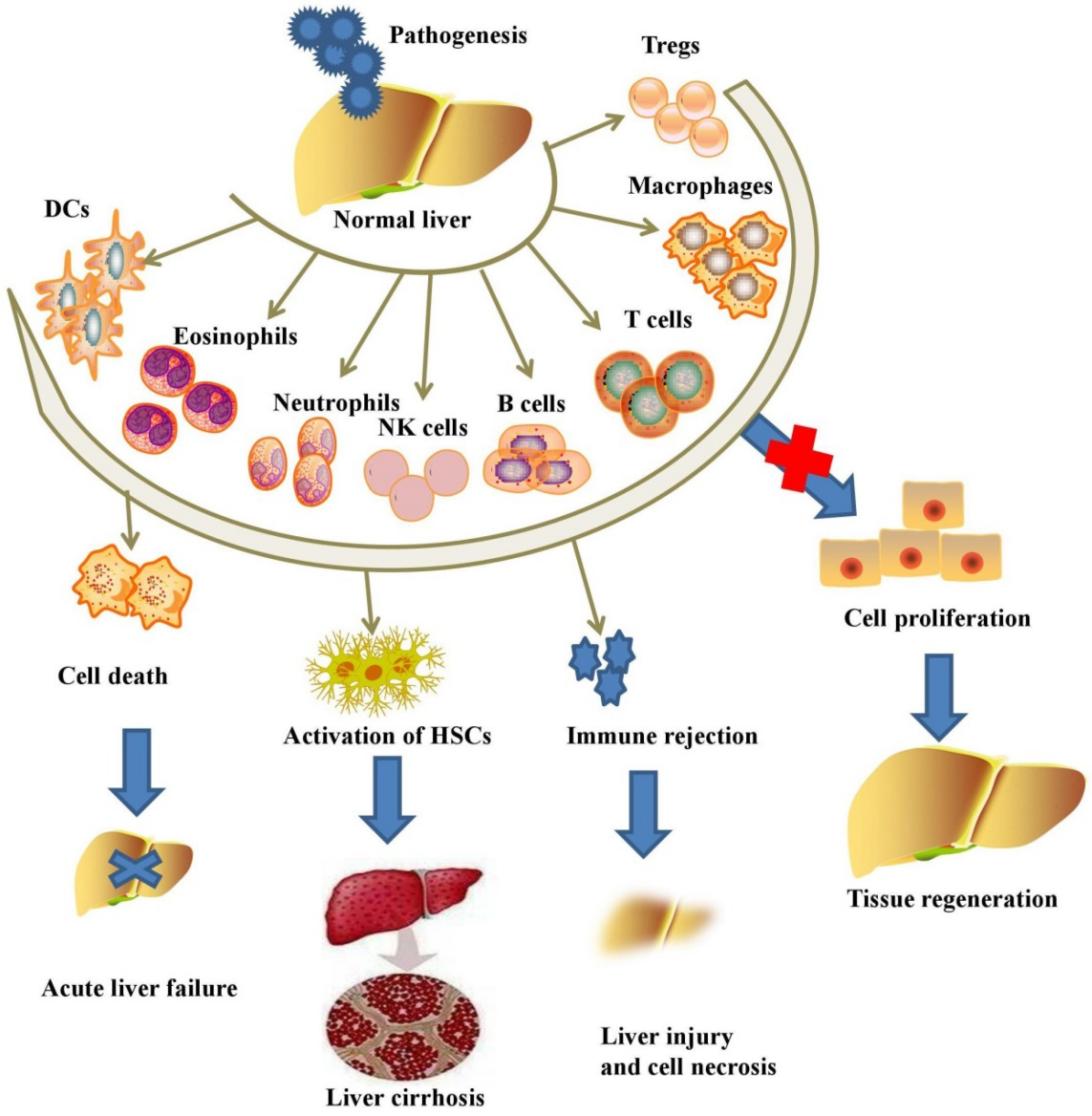
Pathogens initiate the activation of inflammatory immune cells and aggravate acute or chronic liver injury, while the inhibition of immune cells promotes liver regeneration.

In response to liver injury, liver tissues initiate subsequent activation of several subsets of innate immune cells, including macrophages, NK cells, NKT cells, γδT cells, DCs, innate lymphoid cells (ILCs), neutrophils, eosinophils and adaptive immune cells, including T lymphocytes, Tregs, B lymphocytes, Bregs and T helper (Th) cells (**Figure 1**).

Wang et al. demonstrated that a subset of F4/80hiGATA6^+^ macrophages could be recruited from the peritoneal cavity into the liver and further exert their pivotal reparative ability for promoting liver regeneration 24. Furthermore, circulating macrophages are reported to promote the vascularization of liver endothelial cells for liver regeneration 25. Liver-specific macrophages (KCs) represent approximately 20% of the liver nonparenchymal cells and serve as the immune barrier for liver tissue and alert other immune cells through intricate cell-cell interactions and the secretion of cytokines 26. In response to liver injury, KCs subsequently generate a variety of cytokines and chemokines, including TNF-α, CCL2, CCL5, interleukin (IL)-1, and IL-6, recruit other immune cells into liver tissue to promote liver regeneration 27, 28. NK cells are reported to constitute 30%~50% of the intrahepatic lymphocytes in humans, and they play critical roles in controlling bacterial and viral infections in the liver 29. However, other studies debate the protective effects of NK cells in animal models, as they have shown that excessive activation of hepatic NK cells leads to high serum levels of IFN-γ and inhibition of liver regeneration 30, 31. In general, NKT cells can be categorized into pro-inflammatory type I NKT cells and anti-inflammatory type II NKT cells 32, and the two types of NKT cells serve as protective or pathogenic immune cells by inhibiting virus replication or inducing hepatocyte apoptosis and pro-inflammatory cytokine secretion 33-35. However, there is debate about the functions of NKT cells according to a current study. Hosoya et al. showed that NKT cells were not very potent in liver regeneration since CD1d-/- or Jα281-/- mice demonstrated a comparable regeneration rate to wild-type mice after partial hepatectomy 36. γδT cells, which constitute approximately 15%~25% of liver T cells, also serve as a protective or pathogenic immune cell in liver diseases. IFN-γ-producing γδT cells triggered the apoptosis of hepatocytes, while IL-17-producing γδT cells exerted protective effects via inhibition of other immune cells and promotion of the apoptosis of fibrogenic HSCs 37. Partial hepatectomy significantly upregulated the number of IL-17-producing γδT cells, further promoted the secretion of IL-6 and inhibited the secretion of IFN-γ for liver regeneration 38. DCs in liver tissue are divided into two subsets, plasmacytoid DCs (pDCs), which express low levels of MHC-II, and classical DCs (cDCs), which express high levels of MHC-II. Thus, pDCs are limited in presenting antigens, and cDCs are professional antigen-presenting cells 39. Partial hepatectomy dramatically increased the liver DC number and the level of DC-derived TNF-α, thus enhancing the secretion of IL-10 but inhibiting the secretion of IFN-γ from T cells for liver regeneration 40, 41. Hepatic CD49a+ type 1 innate lymphoid cells (ILC1s) limited the recruitment of peripheral NK cells and generated a tolerogenic liver organ to confront various kinds of viral infections 42. Moreover, ILC1s significantly improved the secretion of IL-22 for liver regeneration in response to partial hepatectomy 43. Neutrophils migrate to the injured site of the liver and aggravate liver injury after the generation of reactive oxygen species, pro-inflammatory factors and elastase, while the liver initiates a recovery mechanism after clearing inflammatory neutrophils 44. Activated eosinophils are able to secrete cytokines, cytotoxic granule proteins, enzymes and lipid mediators to cleave pathogens *in vivo* 45. Furthermore, IL-4 secreted from eosinophils was the central factor promoting the proliferation of quiescent hepatocytes in models of partial hepatectomy and toxin treatment, while eosinophil-lacking mice showed a compromised regeneration rate in the liver 46.

In response to IL-6 and other inflammatory factors, T cell differentiation is initiated to augment antiviral adaptive immune responses and mitigate T cell toxicity 47. Tumanov et al. demonstrated that surface lymphotoxin secreted from T cells plays a critical role in liver regeneration, while a deficiency of T cells in mice leads to serious liver injury and reduced DNA synthesis after partial hepatectomy 48. In addition, both Tregs and Th17 cells are subsets of CD4+ Th cells that help to prevent immune response-induced liver injury 49, 50. B cells are critical in liver regeneration after partial hepatectomy since the adoptive transfer of B cells increased lymphotoxin beta production and successfully rescued defective liver regeneration 51. Furthermore, the recruitment of innate-like Bregs and MZ-like B cells from the spleen and peritoneal cavity into liver tissue contributes to the elimination of liver injury 52.

Although external pathogens and immune system imbalances induce liver inflammation and liver injury, the inhibition of immune cell activation contributes to liver repair and liver regeneration.

## MSC transplantation promotes liver regeneration in partial hepatectomy (PH) models

The liver is able to spontaneously regenerate after liver resection, and quiescent hepatocytes and cholangiocytes, which stay in the G_0_ phase of the cell cycle, will undergo proliferation for compensation of lost cells in response to PH 2. Thus, the PH model serves as a standard model for exploring potential mechanisms in liver regeneration. Spontaneous liver regeneration is divided into three stages, the initiation, proliferation, and termination phases after PH, and it is regulated by a complex interactive network consisting of liver cells and extrahepatic organs. In addition, various growth factors, cytokines, hormones, metabolic networks, oxidative stress products, and microRNAs are essential for improving liver regeneration and maintaining hepatic mass 2.

Current studies have shown that MSCs are able to stimulate liver regeneration after surgical resection via promotion of cellular proliferation, downregulation of fat accumulation and paracrine mechanisms. Although MSCs are distributed in multiple tissues, including the brain, thymus, heart, liver, and lung, PH has been shown to induce the migration of MSCs into liver tissue to promote liver regeneration 53. Although a small portion of MSCs are able to engraft in liver tissue, they persist in liver tissue for up to 60 days post-transplantation after 20% liver hepatectomy. Transplanted MSCs undergo hepatogenic differentiation mainly in the periportal area of the injured liver 54. MSC transplantation significantly attenuated metabolic dysfunction and improved liver regeneration by upregulating the proliferation of hepatocytes and sinusoidal endothelial cells, decreasing hepatocyte fat accumulation and decreasing serum levels of IL-6, HGF, and IL-10 in PH-treated mice 55. In addition, MSCs also improved liver regeneration and restored liver function in the PH model partly via mechanistic target of rapamycin (mTOR) signaling and improved lipid *β*-oxidation. In addition, MSCs further activated the STAT3 and Hippo-YAP pathways to improve hepatocyte proliferation for liver regeneration 56. Preoperative resveratrol administration enhanced the homing capacity of MSCs into liver tissue, thus decreasing the expression of TNF-α and IL-6 and improving liver regeneration in PH-treated rats 57. In a 90% PH model, MSCs were proven to improve the glucose metabolism and survival rate of rats with PH-induced ALF via promotion of hepatocyte proliferation through the AKT/GSK-3β/β-catenin pathway 58. Moreover, implantation of bioencapsulated MSCs promoted liver regeneration and increased the survival rate of 90% PH-treated rats from 21% to 91% via induction of hepatogenic differentiation and the secretion of growth factors 59. On the other hand, the MSC-derived secretome promotes liver regeneration, as shown by the increase in the liver-to-body weight ratio and hepatocyte proliferation seen in PH models at an early stage after surgical resection 60.

MSCs not only improved liver regeneration in PH models but also increased hepatocyte proliferation and liver regeneration in PH models with background diseases. Intriguingly, MSCs improved liver function and liver regeneration in repeated PH models after upregulation of HGF expression and attenuation of hepatic vacuolar degeneration 61. In a partial I/R and PH mouse model, human MSCs significantly decreased I/R-induced injury, hindered hepatocellular apoptosis and promoted liver regeneration 62. Although hepatic steatosis significantly inhibited the endogenous regenerative process after PH, MSC transplantation improved liver regeneration and hepatocyte proliferation but decreased hepatocyte apoptosis through paracrine mechanisms 63. MSCs that grow and proliferate will be able to persistently and functionally fulfill the need for regeneration in injured livers after clarification of the related mechanisms in more liver injury models.

## Immunoregulation of MSCs in treating liver diseases

**Figure 2:**
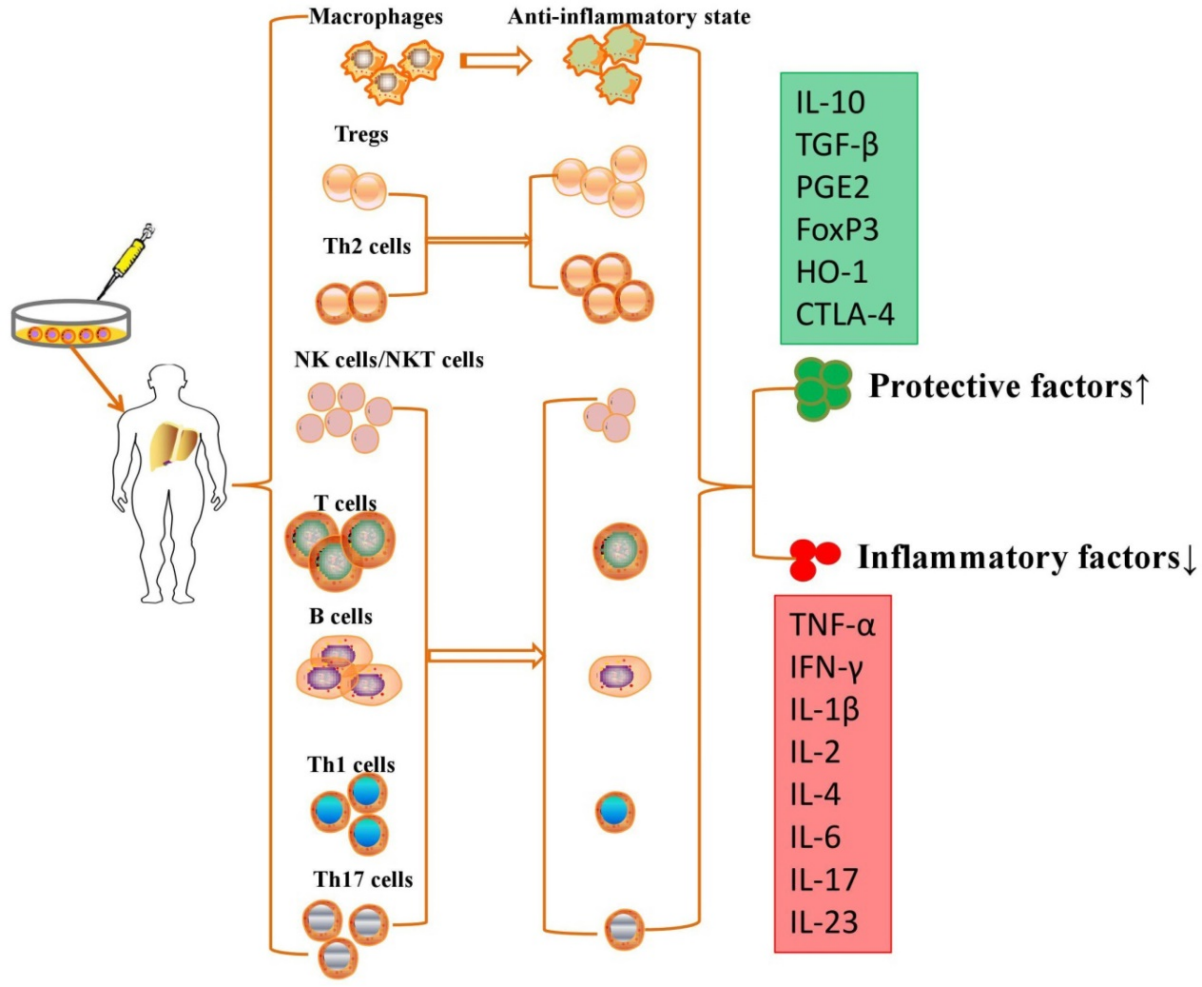
MSCs exert immunomodulatory effects in liver diseases through cell-cell interactions and the secretion of anti-inflammatory factors.

Although no study demonstrated that MSC transplantation participated in immunoregulation in PH models, MSCs have been further proven to repair liver injury by immunoregulation in various liver diseases. MSCs are able to suppress the proliferation, pro-inflammatory cytokine secretion and maturation of immune cells, thus protecting against liver injury in various liver diseases (**Table 1**). MSCs exert immunomodulatory effects in liver diseases through cell-cell interactions and the secretion of anti-inflammatory factors (**Figure 2**).

Intravenously infused MSCs were phagocytosed in lung tissue, and these multipotent cells migrated from the lungs to other body sites to exert immunomodulatory effects via secretion of programmed death ligand-1 and IL-10 64. MSCs are able to switch macrophages from a pro-inflammatory state to an anti-inflammatory state after secretion of prostaglandin E2 (PGE2), TNF-α, stimulated gene/protein 6, IL-6, and indolamine 2,3-dioxygenase (IDO) 65. On the other hand, MSCs also migrate into liver tissue and decrease the levels of alanine aminotransferase and proinflammatory cytokines and the degree of liver inflammatory cell infiltration by restraining the activation of NK cells and decreasing the expression of natural killer group 2, member D (NKG2D) in liver 66. Aggarwal et al. showed that MSCs significantly inhibit the secretion of TNF-α by mature type 1 DCs and decrease IFN-γ secretion by Th1 and NK cells; however, MSCs significantly upregulated the proportion of Tregs, increased the secretion of IL-10 by mature DC2s and upregulated the secretion of IL-4 by Th2 cells 67. They effectively inhibited T cell proliferation to induce an immunotolerant state 68 and promoted the development and polarization of naive T cells into Tregs 69. MSCs have been reported to suppress the production and secretion of antibodies and the proliferation of activated B lymphocytes in an IDO-dependent manner 70. However, the immunomodulatory properties of MSCs vary according to the specific liver disease, and these multipotent cells are molded into an anti-inflammatory role for liver regeneration and immune tolerance.

### Immunoregulation of MSCs in treating ALF

ALF is a life-threatening disease that progresses within 26 weeks and is accompanied by abundant hepatic necrosis, coagulation dysfunction, and mental alterations in patients without preexisting liver cirrhosis. Commonly, chemicals, metabolic substances, infectious pathogens and surgery are used to generate models with acute liver injury or liver failure 71. The immunoregulation of MSCs for treating ALF is still under exploration in chemically treated animal models.

MSCs significantly decreased the incidence of cell death, hepatocyte cytoplasmic vacuolization and macrophage infiltration, thus improving liver histopathology and prolonging the survival time of ALF mice 72. MSCs significantly attenuated acute liver injury by reducing the total number of IL-17-producing NKT cells without influencing the number of IL-17-producing neutrophils, CD4+ T lymphocytes, and CD8+ T lymphocytes in ALF mice 73. However, Zhang et al. demonstrated that MSCs significantly decreased liver CD4+ T cell infiltration, CD4+ T cell activation and the total number of Th1 cells, which was accompanied by the induction of Tregs and regulatory DCs in the liver to attenuate liver injury 74. Acute liver injury and inflammation recruited the injected MSCs into injured liver sites to suppress the activity of intrahepatic TNF-α-, IFN-γ-, and IL-4-producing NKT cells and increase the serum level of IL-10, which subsequently ameliorated liver damage in a time- and dose-dependent manner 75. In addition, MSCs also inhibited the accumulation of CD11b+, Gr-1+, and F4/80+ cells but increased the number of Tregs in the liver of mice with acute hepatitis 76, 77.

In addition to transplantation of MSCs alone, ALF may be synergistically eliminated by enhancing growth-related gene expression to block the effect of inflammatory factors. For example, IL-35-overexpressing MSCs inhibited the secretion of IFN-γ in liver mononuclear cells via regulation of the JAK-1-STAT-1/STAT-4 signaling pathway, thus protecting against hepatocyte apoptosis in acute fulminant hepatitis 78. To this end, MSCs transformed the body into an anti-inflammatory state after upregulating anti-inflammatory immune cells in the serum and liver, thus reducing the hepatocyte apoptosis rate and promoting the liver regeneration process in ALF models.

### Immunoregulation of MSCs in treating NAFLD and liver fibrosis

NAFLD happens in individuals with no history of alcohol abuse and is accompanied by the accumulation of excessive fat in the liver. The development of NAFLD is attributed to metabolic factors and genetics, and the disease can progress into nonalcoholic steatohepatitis, hepatic fibrosis, liver cirrhosis and hepatoma 79.

Immune cells, including macrophages, NK cells, and T cells, participate in the progression of NAFLD and serve as potential therapeutic targets. Diminishing the resident KCs and recruited bone marrow-derived macrophages led to systemic insulin resistance and NASH, which indicates the importance of macrophages in prohibiting the development of NAFLD 80. Cur et al. demonstrated that the initiation of hepatocyte apoptosis in NASH patients activated NK cells in a NKG2D-dependent manner; these NK cells contribute to reversing liver fibrosis by inhibiting and killing HSCs 81. In addition, apoptosis of liver NKT cells also contributes to insulin resistance and steatosis in NAFLD 82. MSC transplantation decreased high‑fat diet‑induced weight gain, steatosis, lobular inflammation and liver fibrogenesis in mice with NAFLD via inhibition of splenic CD4+IFN-γ+ and CD4+IL-6+ T lymphocyte differentiation, while it had no effect on the activity of CD4+IL-17+ T lymphocytes 83. Seki et al. demonstrated that MSCs engrafted into the liver and restored albumin secretion in a NASH model by reducing the number of intrahepatic infiltrating CD11b+ and Gr-1+ cells and the ratio of CD8+/CD4+ cells 84.

In addition to NAFLD, other factors, including the hepatitis virus, alcohol consumption, cholangitis and autoimmune hepatitis, result in the accumulation of aberrant myofibroblasts and extracellular matrix in the liver and subsequent liver fibrosis and are associated with poor prognosis 85. Monocytes, the precursors of fibrocytes, macrophages and DCs, are vital to the progression of liver fibrosis. IL-4 and IL-13 derived from activated CD4+ T cells can stimulate the differentiation of fibrogenic myeloid cells and macrophages in liver tissue. Moreover, the secretion of IL-17A from Th17 cells further activates myofibroblasts directly and indirectly. In addition to these fibrogenic cells, both Tregs and macrophages are fibrogenic or fibrolytic during fibrosis progression and fibrosis reversal 86. However, KCs can generate matrix metalloproteinases and promote matrix degradation to resolve liver injury and fibrosis 87, 88. MSCs significantly attenuated CCl_4_-induced liver fibrosis by decreasing the percentage of Th17 cells and increasing CD4+IL-10+ T cells and immunosuppressive factors including IL-10, IDO and kynurenine 89. MSC transplantation reversed liver fibrosis by downregulating the serum levels of IL-17, IL-2 and IL-6 and the expression of IL-17A and IL-17RA in the liver, which was accompanied by downregulation of the expression of STAT3, p-STAT3, P-SMAD3 and TGF-βR2 90.

In addition, MSCs also improved liver function and clinical symptoms in patients with liver fibrosis via attenuation of pro-inflammatory responses and improved anti-inflammatory responses. Administration of MSCs significantly downregulated the levels of IL-6 and TNF-α but upregulated the level of IL-10 in patients with hepatitis B virus-induced decompensated liver cirrhosis after 2 and 4 weeks. Moreover, MSCs also increased the levels of CD4+ T cells and Tregs but decreased the levels of CD8+ T cells and B cells. Although MSCs did not attenuate the development of liver failure compared with standard treatments, they remarkably decreased the level of aspartate aminotransferase and improved the levels of albumin and total bilirubin and the prothrombin time 12. When added to entecavir treatment, autologous MSCs further improved liver function in hepatitis B virus-induced liver cirrhosis patients. Moreover, MSCs significantly increased the levels of Tregs and Foxp3 but decreased the levels of Th17 cells and retinoic acid receptor-related orphan receptor gamma t (RORγt). MSCs also improved the serum TGF-β level but decreased the levels of serum IL-17, TNF-α, and IL-6 91.

Patients with primary biliary cirrhosis (PBC), a kind of prototypical autoimmune disease, have a higher number of autoreactive liver-infiltrating CD4+ T cells and CD8+ T cells in their livers than in their blood 92, 93. In addition, activated NKT cells enhance the death of biliary epithelial cells, which leads to the progression of PBC 94. Intriguingly, MSC transplantation significantly alleviated symptoms of fatigue and pruritus in most patients who responded only partially to ursodeoxycholic acid treatment 95. Moreover, allogeneic MSC transplantation significantly increased the level of CD4+Foxp3+ Tregs in peripheral blood and in lymph nodes in a PBC animal model, thus inhibiting the systemic immune response and enhancing liver recovery from liver inflammation in PBC mice 96. As we know, few studies have focused on the potential mechanisms of MSC transplantation in the attenuation of liver injury in PBC, and it is necessary to expand related studies for clinical applications.

### Immunoregulation of MSCs in recipients who have undergone LT

LT is the standard therapy for patients with end-stage liver diseases, while discontinuation of immunosuppression when the native liver regenerates after LT may lead to spontaneous rejection and atrophy of the allogeneic liver graft 97. Acute rejection after LT is usually treated with large doses of immunosuppressants with severe toxicity and side effects, so it is imperative to find a safe and effective method for preventing rejection in individuals with LT. In this regard, immunomodulatory cell therapy is an innovative approach to complement standard pharmacotherapy in LT recipients. Since MSCs exert immunomodulatory and regenerative effects *in vivo*, they effectively prolong organ allograft survival and reduce the side effects of transplantation and concomitant therapy.

Following LT, the initiation of liver graft rejection is accompanied by upregulation of peripheral blood Th1 cells, while the upregulation of Th2 cells indicates long-term acceptance or tolerance of transplanted livers 98. The infusion of MSCs upregulated the levels of Th2 cells and Tregs but decreased Th1 and Th17 cells, consequently reducing the expression of IL-2, IL-6, IL-17, IL-23, IFN-γ and TNF-α and increasing the concentrations of IL-10 and TGF-β in rats after LT. To this end, MSC administration protected against liver dysfunction and hepatic apoptosis and significantly decreased the acute rejection rate and improved the survival rate in LT rats 99. In contrast to the side effects seen with general therapy with conventional immunosuppressive agents, Shi et al. observed no side effects of MSC transplantation in LT patients, and they found that MSC infusions markedly downregulated alanine aminotransferase levels and improved allograft histology throughout the 12-week follow-up. However, they argued that MSCs effectively increased the ratio of Tregs and Th17 cells and the related cytokines (TGF-β1 and PGE2) after 4 weeks 100. In addition, MSCs prolonged the survival of mice that had undergone LT by increasing the expression of FoxP3 and CTLA-4 and decreasing the proliferation of CD4+ T cells and the allospecific CTL activity of CD8+ T cells 101. However, the administration time of MSCs will influence the effects in attenuating liver graft rejection. Xia et al. highlighted that only MSC administration early after LT prolonged the survival time of rats and inhibited the typical LT-related acute graft versus host disease (aGVHD) compared with MSC transplantation from day 8 to day 14 in rats 102.

A variety of key genes are overexpressed in MSCs to enhance their immunoregulatory capacities and improve LT prognosis. After coculture with KCs, MSCs downregulated the levels of pro-inflammatory cytokines, such as IL-1β and IL-6 while upregulating the levels of anti-inflammatory cytokines, such as TGF-β, IL-4, PGE2 and IL-10. Moreover, PGE2-overexpressing MSCs and TGF-β1-overexpressing MSCs more significantly increased the allograft tolerance and improved the survival of rats after LT 103, 104. Foxp3-overexpressing MSCs generated a state of Treg-dependent tolerance and induced donor-specific allograft tolerance in a contact-dependent mechanism 105. Transplantation of heme oxygenase (HO)-1-overexpressing MSCs significantly decreased the rejection rate while increasing the survival time of LT rats by increasing the levels of peripheral Tregs and decreasing the levels of NK cells in liver tissue 106, 107. Furthermore, overexpression of HO-1 or IL-10 in MSCs significantly increased the levels of anti-inflammatory cytokines (IL-10 and TGF-β) and decreased the levels of proinflammatory cytokines (IL-2, IL-6, IL-17, IL-23, TNF-α, and IFN-γ) in LT recipients 106-108. Gene modification of MSCs will further contribute to the inhibition of LT rejection since multiple cytokines participate in MSC immunoregulation *in vivo*. However, it is worth mentioning that gene modification may induce epigenetic changes or gene mutations in MSCs, potentially leading to tumor generation *in vivo*.

## Conclusions and Future Directions

Immune cell infiltration is an essential step leading to liver injury, which is accompanied by hepatocyte apoptosis, HSC activation, uncontrolled wound-healing pathophysiology, formation of intrahepatic scar tissue and tumorigenesis. As previously reported, MSCs are able to migrate toward injured tissues, undergo hepatogenic differentiation, inhibit inflammatory factor release and enhance the proliferation of liver cells *in vivo*. Although the application of MSCs is extensive in inflammation-related liver diseases, the elucidation of the molecular mechanisms underlying the interactions between MSCs and immune cells can further help us to develop new MSC-based therapies for treating liver diseases. MSCs inhibit the activation of proinflammatory immune cells but enhance the activation of anti-inflammatory immune cells to generate an immunotolerant microenvironment in liver tissue. It is worth mentioning that gene modification of these cytokines may help to improve the therapeutic effects of MSC transplantation in liver diseases but could lead to potential tumorigenesis. As we have discussed, MSC transplantation is an effective anti-inflammatory strategy for eliminating acute or chronic liver injury and has great potential for application in liver regenerative medicine.

## Figures and Tables

**Table 1:** MSCs exert immunoregulation in the treatment of ALF, NAFLD, and liver fibrosis patients and recipients of LT.

Modification	MSC source	Model	Pathogenesis	Mechanism	Effect	Ref.
N/A	Bone marrow	Mouse	CCl4	IL-17-producing NKT cells↓; FoxP3+IL-10+ NKT cells ↑	Liver injury↓; liver inflammatory cell infiltration↓	73
N/A	Bone marrow	Mouse	TAA	Incidence of cell death↓; hepatocyte cytoplasmic vacuolization↓; macrophage infiltration↓	Liver histopathology↓; survival time of ALF mice↑	72
N/A	Bone marrow	Mouse	ConA	NKT activation↓; lymphocyte proliferation↓	Liver damage↓	109
N/A	Bone marrow	Mouse	ConA	TNF-α+ NKT cells↓; IFN-γ+ NKT cells↓; IL-4+ NKT cells↓	Liver damage↓	75
N/A	Adipose tissue	Mouse	ConA	CD11b+Gr-1+ F4/80+ cells↓	Serum ALT and LDH activity↓; hepatocyte necrosis↓	76
Overexpression of IL-35	Adipose tissue	Mouse	ConA	IFN-γ+ liver mononuclear cells↓	Hepatocyte apoptosis↓; survival rate of ALF mice↑	78
N/A	Bone marrow	Mouse	α-GalCer	TNF-α+ NKT cells↓; IFN-γ+ NKT cells↓; IL-4+ NKT cells↓	Liver damage↓	75
N/A	Bone marrow	Mouse	α-GalCer	IL-17+ NKT cells↓; FoxP3+IL-10+ NKT cells ↑	Liver injury↓; liver inflammatory cell infiltration↓	73
N/A	Bone marrow	Mouse	α-GalCer	CD4+ cells↓; IL10-producing CD4+CD25+FoxP3+ Tregs↑; IL-6- and TNF-α-producing inflammatory B cells↓; migration of inflammatory cells from the spleen into the injured livers↑	Serum aminotransferase↓; tissue necrosis↓	110
N/A	Bone marrow	Mouse	LPS	Liver CD4+ T cell infiltration↓; CD4+ T cell activation↓; total number of Th1 cells↑; induction of Tregs and regulatory DCs in the liver↑; DC-induced Treg differentiation↑	Liver functions↑; survival time of ALF mice↑	74
N/A	Bone marrow	Mouse	Methionine-choline deficient diet	Activation of CD4+IFN-γ+ lymphocytes↓; activation of CD4+IL-6+ lymphocytes↓	Weight loss↓; hepatic lipid peroxidation↓; hepatic steatosis↓; hepatic lobular inflammation↓; liver fibrogenesis↓	83
N/A	Adipose tissue	Mouse	Atherogenic high-fat diet	The number of intrahepatic infiltrating CD11b+ and Gr-1+ cells and the ratio of CD8+/CD4+ cells↓	Engraftment into the liver↑; albumin secretion↑; liver fibrosis↓	84
N/A	Umbilical cord	Human	Hepatitis B virus	Levels of CD4+ cells and Tregs↑; levels of CD8+ cells and B cells↓; levels of IL-6 and TNF-α↓; level of IL-10↑	Level of aspartate aminotransferase↓; levels of albumin and total bilirubin and the prothrombin time↑	12
N/A	Bone marrow	Human	Hepatitis B virus	Tregs↑; Foxp3↑; Th17 cells↓; RORγt↓	Liver function↑;	91
N/A	Bone marrow	Mouse	CCl_4_	Th17 cells↓; serum IL-17 level↓; CD4+IL-10+ T cells↑; levels of IL-10, IDO and kynurenine↑	Liver functions↑; liver fibrosis↓	89
N/A	Bone marrow	Rat	CCl_4_	Expression of IL-17, IL-2 and IL-6 in serum and expression of IL-17A and IL-17RA in liver↓; expression of STAT3, p-STAT3, P-SMAD3 and TGF-βR2↓	Liver functions↑	90
N/A	Bone marrow	Mouse	Polyinosinic-polycytidylic acid sodium	CD4+Foxp3+ Tregs in peripheral blood and in lymph nodes↑; serum TGF-β1 ↑; IFN-γ ↓	Liver recovery↑	96
N/A	Bone marrow	Rat	LT	Levels of Th2 cells and Tregs↑; number of Th1 and Th17 cells↓	Acute rejection rate ↓; levels of liver enzymes and hepatic apoptosis↓; survival rate of LT mice↑	99
N/A	Umbilical cord	Human	LT	Ratio of Tregs and Th17 cells↑; levels of TGF-β1 and PGE2↑	Alanine aminotransferase level↓; allograft histology↑	100
N/A	Bone marrow	Rat	LT	Proliferation of CD4+ T cells↑; allospecific CTL activity of CD8+ T cells↓	Survival time of mice that had undergone LT↑	101
N/A	Bone marrow	Rat	LT	Treg ratios in peripheral blood↑; levels of FoxP3-positive cells↑;	Survival time of rats↑; typical LT-related acute graft versus host disease↓	102
Overexpression of PGE2	Bone marrow	Rat	LT	KC reprogramming↑; levels of TNF-α and PGE2↑	Allograft tolerance↑; survival rate of rats after LT↑	103
Overexpression of TGF-β1	Bone marrow	Rat	LT	CD4+Foxp3+Helios- induced Tregs↑; Th17 cells↓; immunosuppressive effects in local liver grafts↑	Acute rejection rate after LT↓; mortality of rats after LT↓; survival of rats after LT↑	104
Overexpression of HO-1	Bone marrow	Rat	Reduced-size LT	Activity of NK cells↓; proportion of regulatory T cells (Tregs)↑	Median survival time↑; rejection activity index↑	106
Overexpression of HO-1	Bone marrow		LT	Levels of peripheral Tregs and anti-inflammatory cytokine (IL-10 and TGF-β) levels↑; NK cell activity and proinflammatory cytokine (IL-2, IL-6, IL-17, IL-23, TNF-α, and IFN-γ) levels↓	Survival time of rats after LT↑; apoptosis rate of hepatocytes and degree of rejection↓	107
Overexpression of IL-10	Bone marrow	Rat	LT	Expression of cytokines (IL-17, IL-23, IL-6, IFN-γ and TNF-α)↓; RORγt↓; expression of IL-10 and TGF-β1↑; FoxP3↑	Mean survival time of rats after LT↑	108
Overexpression of Foxp3	Bone marrow	Rat	LT	Proliferation of allogeneic CD4+ T cells↓; level of programmed death ligand 1↑; CD4+CD25+Foxp3+ Tregs↑	Donor-specific allograft tolerance↑	105

## Abbreviations

KCs: Kupffer cells
HSCs: hepatic stellate cells
DCs: dendritic cells
NK: natural killer
NKT: natural killer T
LT: liver transplantation
MSC: mesenchymal stromal cell
Tregs: regulatory T cells
Bregs: regulatory B cells
ALF: acute liver failure
NAFLD: nonalcoholic fatty liver disease
ILCs: innate lymphoid cells
TNF-α: tumor necrosis factor-α
IL: interleukin
IFN: interferon
pDCs: plasmacytoid DCs
cDCs: classical DCs
NASH: nonalcoholic steatohepatitis
PGE2: prostaglandin E2
IDO: indolamine 2,3-dioxygenase
NKG2D: natural killer group 2, member D
CCl_4_: carbon tetrachloride
TAA: thioacetamide
ConA: concanavalin A
α-GalCer: α-galactosylceramide
LPS: lipopolysaccharide
iNOS: inducible nitric oxide synthase
FoxP3: forkhead box protein 3
RORγt: retinoic acid receptor-related orphan receptor gamma t
PBC: primary biliary cirrhosis
aGVHD: acute graft versus host disease
HO: heme oxygenase
